# Naturally occurring canine sarcomas: Bridging the gap from mouse models to human patients through cross-disciplinary research partnerships

**DOI:** 10.3389/fonc.2023.1130215

**Published:** 2023-03-23

**Authors:** Marika Klosowski, Laurel Haines, Lauren Alfino, Alexandra McMellen, Michael Leibowitz, Daniel Regan

**Affiliations:** ^1^ Flint Animal Cancer Center, College of Veterinary Medicine and Biomedical Sciences, Colorado State University, Fort Collins, CO, United States; ^2^ Department of Microbiology, Immunology, and Pathology, College of Veterinary Medicine and Biomedical Sciences, Colorado State University, Fort Collins, CO, United States; ^3^ Center for Cancer and Blood Disorders, Children’s Hospital Colorado, Aurora, CO, United States

**Keywords:** canine (dog), sarcoma, osteosarcoma, comparative oncology, immunotherapy

## Abstract

Fueled by support from the National Cancer Institute’s “Cancer Moonshot” program, the past few years have witnessed a renewed interest in the canine spontaneous cancer model as an invaluable resource in translational oncology research. Increasingly, there is awareness that pet dogs with cancer provide an accessible bridge to improving the efficiency of cancer drug discovery and clinical therapeutic development. Canine tumors share many biological, genetic, and histologic features with their human tumor counterparts, and most importantly, retain the complexities of naturally occurring drug resistance, metastasis, and tumor-host immune interactions, all of which are difficult to recapitulate in induced or genetically engineered murine tumor models. The utility of canine models has been particularly apparent in sarcoma research, where the increased incidence of sarcomas in dogs as compared to people has facilitated comparative research resulting in treatment advances benefitting both species. Although there is an increasing awareness of the advantages in using spontaneous canine sarcoma models for research, these models remain underutilized, in part due to a lack of more permanent institutional and cross-institutional infrastructure to support partnerships between veterinary and human clinician-scientists. In this review, we provide an updated overview of historical and current applications of spontaneously occurring canine tumor models in sarcoma research, with particular attention to knowledge gaps, limitations, and growth opportunities within these applications. Furthermore, we propose considerations for working within existing veterinary translational and comparative oncology research infrastructures to maximize the benefit of partnerships between veterinary and human biomedical researchers within and across institutions to improve the utility and application of spontaneous canine sarcomas in translational oncology research.

## Introduction

Sarcomas are a heterogeneous group of neoplasms which arise from mesenchymal cells within tissues derived from the embryonic mesoderm. Sarcomas are rare in people and account for only about 1% of all cancers, though pediatric patients are disproportionately burdened by this tumor type as sarcomas represent greater than 20% of pediatric solid tumors (1). Sarcomas are broadly subdivided into categories of bone and soft tissue sarcomas, however there are over 70 histologically and genetically distinct sarcoma subtypes recognized, and the incidence of each of these unique sarcoma subtypes is substantially lower. Thus, patient recruitment for clinical trials in rarer sarcomas remains a significant barrier to the development of new therapies. Despite the rarity of sarcomas, sarcoma research has historically made an outsized contribution to our understanding of the fundamental biology of cancer and laid the groundwork for remarkable advances in cancer treatment including immunotherapy and personalized small-molecule inhibitor therapy (2). Still, treatment advances for sarcoma patients have lagged behind more common cancer types, particularly with regard to the paradigm-shifting cell-based therapies recently developed for hematologic malignancies. Novel therapies for treatment-refractory, relapsed, and metastatic sarcomas are lacking and overall survival remains quite poor for these patients.

Animal models are essential for testing new interventions for these rare cancers at a meaningful and time-efficient scale. Immune competent and immunodeficient murine models are often utilized in pre-clinical sarcoma research and have provided the rationale for novel therapies. While studying experimentally induced tumors in mouse models is cost-effective, accessible, and affords a high degree of reproducibility, the limitations of these models are becoming increasingly apparent. It is estimated that 85% of novel drugs which show promise in preclinical testing will fail in early clinical trials. The failure rate for cancer drugs is even greater, with as few as 8% of novel cancer drugs translated successfully from preclinical animal models to human clinical trials (3–5). The statistics surrounding sarcoma drug development are even more discouraging, with estimates suggesting only 5% of Phase I/II sarcoma trials make it to Phase III studies (6). This unacceptable outcome is in part driven by the fact that many of the drugs tested in sarcoma patients are drugs originally developed, tested, or approved for other cancer types (6). Most of these failures are attributed not to drug safety concerns, but rather to lack of efficacy (7), suggesting that *in vivo* preclinical testing as currently performed is poorly predictive of clinical efficacy. Given the complex interplay of genetic, biological, and environmental factors influencing disease phenotype and outcomes in cancer patients, it is unsurprising that preclinical rodent models are limited in their ability to predict treatment efficacy in humans and it is apparent that incorporation of additional representative animal models is desperately needed to enhance the efficiency and success of cancer drug development.

**Figure 1:**
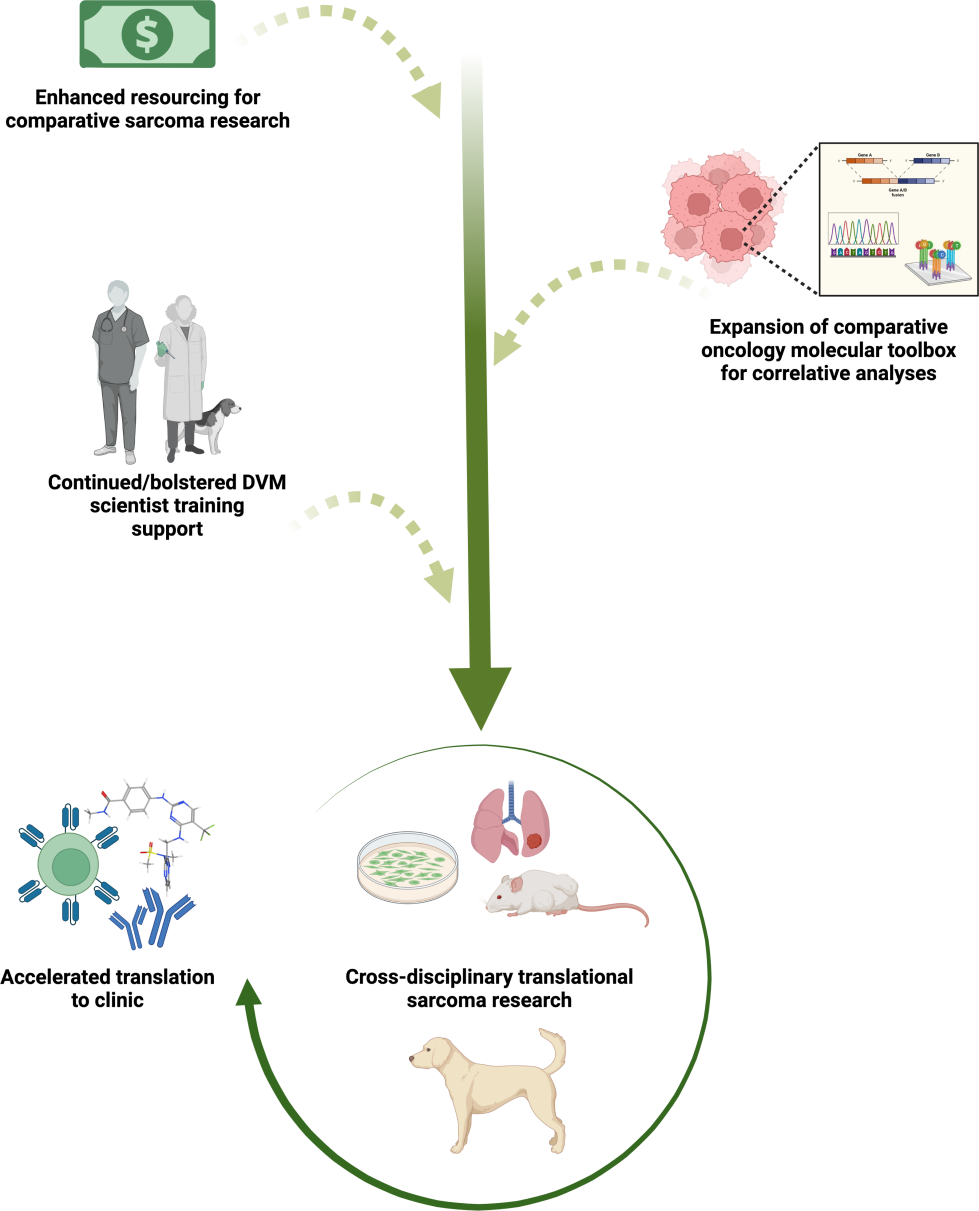
Sustaining and bolstering existing comparative oncology research infrastructure for continued acceleration of sarcoma therapeutic discovery and translation to clinical trial evaluation. Increasing comparative oncology research funding and industry investment in development of species-specific molecular tools, paired with veterinary and medical collaborative sarcoma research, will enhance an already effective pathway for more predictive vetting of novel preclinical agents and movement into human clinical trials. Evaluation of small molecule immunotherapies, novel molecular-targeted agents, monoclonal/bi-specific antibodies, and adoptive cell therapies alone and in combination are likely to be especially informative to the field. Created with BioRender.com.

Pet dogs with spontaneously occurring cancers have received increasing attention as a promising model to bridge the translational gap between rodent tumor models and human clinical trials. Cancer and associated complications are the leading cause of death in most dog breeds and mixed breed dogs in North America, and their large size, relative outbreeding, and shared environmental exposures with their human caregivers are advantageous for modeling human cancers. In contrast with their rare incidence in people, sarcomas comprise a much larger proprotion of malignant tumors in dogs (8). The increased incidence of sarcomas in dogs provides both an impetus for veterinary sarcoma research and a comparative research opportunity. Canine sarcomas share many genetic, biologic, and histologic features with their human counterparts. Most importantly, canine spontaneous cancer models retain the complexities of naturally occurring therapeutic resistance, spontaneous metastasis, and tumor-host immune interactions seen in human patients. Here, we provide an overview of translationally relevant spontaneously occurring canine sarcomas and offer perspectives on optimal application of these models in current and future translational research (**Figure 1**), as well as considerations for expanding translational research training and facilitating greater inclusion of veterinary clinician-scientists into cross-disciplinary cancer research teams.

## Naturally occurring canine sarcomas

### Osteosarcoma

Osteosarcoma (OS) is the most common primary malignant bone tumor in both dogs and people (1, 8). However, it remains a rare disease in people and accounts for approximately 2% of all childhood cancers, and less than 1% of adult cancers (1). Based on recent OS incidence estimates, less than 1000 new cases of OS are diagnosed yearly in the United States (9), and the rarity of this tumor has greatly limited its study in human patients. In contrast, canine osteosarcoma accounts for about 5% of all canine cancers and the incidence of OS in dogs is estimated to be 10-30 times higher than in people (though incidence rates vary across dog breeds), with at least 10,000 new cases diagnosed in the US each year by conservative estimates (10–12). Thus, the inclusion of dogs with OS can greatly expand the available patient populations for translational OS research.

Appendicular OS arising from the metaphysis of long bones is the most common and best researched form in both people and dogs and has a remarkably similar clinical presentation in both species, including a similar bimodal age distribution (12, 13–15). However, notable differences in affected sites are seen between these species, with a majority of canine OS occurring in the thoracic limbs while OS in people most commonly affects the long bones of the pelvic limb (13, 15). Thoracic limb OS is associated with an increased risk of metastasis and subsequently a more rapid and aggressive disease course, and thus this apparent site predilection makes dogs a beneficial model for studying the biology and treatment of OS within the context of the more aggressive disease course seen with thoracic limb osteosarcoma (13, 15, 16). The histological subtypes of OS observed in dogs also closely parallel those observed in people (17–20), and the most common OS subtypes in people are seen with similar prevalence in the dog (13, 19–21). However, the prognostic importance of OS histological subclassification in dogs is unknown and it is not a feature included in the histological grading of canine OS (22). Additionally, reported cases of juxtacortical (periosteal and parosteal) OS in dogs are rare, and it is not known whether these surface OS subtypes carry an improved prognosis as is documented in people (19, 23, 24). Retrospective analyses indicate that metastatic disease in both human and canine OS results in worse clinical outcomes. Strikingly, over 80% of patients of both species are presumed to have micrometastases at diagnosis, despite a minority of these patients having detectable metastatic disease (25, 26). Thus, prevention and treatment of metastatic disease represents a critical area for utilization of canine OS models to improve survival rates which have been largely stagnant over the past several decades for both species.

The molecular landscape of canine OS has been relatively well characterized compared with other canine sarcomas. Comparative genomic analysis of OS from dogs and people consistently identifies striking similarities between these tumors, with one study finding them indistinguishable by global gene expression signature (27). The genomic structural complexity in canine OS appears to parallel that of human OS, with both patterns of localized somatic hypermutation consistent with kataegis as well as complex chromosomal rearrangements suggestive of chromothripsis identified in canine OS tumors (28). Furthermore, multiple studies corroborate genomic aberrations in both human and canine OS converging on key shared tumorigenic pathways, with both tumors characterized by a high prevalence of TP53 mutations (28–31) and hyperactivated PI3K/MAPK signaling (28, 30–33).

While further exploration is warranted (and ongoing), the genetic similarities between the canine and human tumors suggest rich opportunities for utilization of canine OS models in the development of new prognostication methods and personalized, targeted therapeutics. Indeed, comparison of the genetic signatures of canine and human OS have led to identification of a pair of genes (CXCL8 and SLC1A3) for which high expression in human tumors was linked with poorer clinical outcome (27). The clinical significance of one of these genes, CXCL8, on lung metastatic OS progression and response to macrophage-targeted immunotherapeutic intervention was also subsequently verified in two independent studies in people and dogs, respectively (34, 35). Unlike many rodent tumor models, dogs with OS also offer an opportunity to investigate tumor-immune interactions within an immunocompetnt host, and because of this have proven instrumental in identifying the mechanisms by which OS may modulate local and global anti-tumor immune responses to promote progression and metastasis (36–38). Additionally, though the general principles of treatment protocols are similar across these species (39–42), neoadjuvant or multi-drug adjuvant chemotherapeutic protocols are infrequently pursued in dogs due to quality-of-life concerns in the face of questionable clinical benefit. This makes dogs with OS a unique patient population in which to assess immunologic targets and therapies in the neo-adjuvant setting where they may demonstrate greater prognostic and therapeutic benefit.

### Vascular sarcomas

Angiosarcoma (AS) refers to a group of sarcomas which exhibit differentiation toward vascular or lymphatic endothelial cells. These are among the rarest of the sarcoma types in humans, comprising less than 1% of all soft tissue sarcomas (43). The incidence of hemangiosarcoma (HSA), the canine analog of AS, is estimated to be 25 to 100 times higher than in people, and HSA accounts for 5% of all non-cutaneous primary malignant neoplasms in dogs (15, 44). However, it should be noted that while the human angiosarcoma term encompasses multiple vascular sarcomas including lymphangiosarcoma, these tumors are still denoted separately in veterinary literature with hemangiosarcoma commonly described while only 30 cases of lymphangiosarcoma have been reported since this tumor was first documented in the dog in 1981 (45, 46). The extremely rare and heterogeneous nature of AS has made researching this tumor in human patients particularly challenging, and treatment options for people with AS remain limited and patient mortality high. The much higher incidence of a similar tumor in dogs highlights an important opportunity for comparative research with the potential to generate novel treatment strategies.

The clinical presentations of AS and HSA are similar with both tumors diagnosed predominantly in older patients (43, 47). However, the distribution of affected sites differs between dogs and people, likely in part due to differences in environmental exposures (44). UV light exposure and radiation treatment for other cancer types (particularly breast cancer) are well-documented risk factors for AS, and correspondingly this neoplasm most frequently presents in the cutaneous and subcutaneous tissues of the head, neck, and breast (48, 49). In contrast, visceral HSA affecting the spleen or right cardiac atrium is the most common presentation in the dog (47), though UV-light exposure has also been implicated in the development of cutaneous HSA in this species (50, 51). The pathologic features of HSA closely parallel those of AS, with neoplastic cells characterized by variable vascular differentiation and expression of endothelial-associated markers CD34, CD31, and von Willebrand’s factor (52–57). A histologic grading scheme has not been established for canine HSA, but tumor stage and primary site appear to be useful grade-independent prognostic factors for both species (58–62).

AS and HSA exhibit similarly aggressive biologic behavior with highly infiltrative and invasive patterns of local growth and a high risk of metastasis contributing to the poor clinical outcomes seen in both species (44, 48–50). The 1-year survival rate in dogs who underwent surgical resection followed by adjuvant chemotherapy is only about 10% (63), and in humans the five-year survival rate is only about 35% to 40% despite multi-modal standard-of-care treatment with surgery followed by adjuvant radiotherapy and chemotherapy (64–66). Though shorter survival times in dogs with HSA likely also reflect differences in treatment intent and stage at diagnosis, as in OS, the more rapid disease course of HSA in this species allows for the assessment of clinical endpoints within an accelerated timeline.

The cellular origins of both AS and HSA remain incompletely understood, but recent genomic profiling of canine HSA has driven paradigm shifts regarding the histogenesis of this tumor. Though classically thought of as arising from transformed endothelial cells, these data suggest a pluripotent bone marrow progenitor cell of origin for HSA (67, 68). While ontogenic molecular studies have been comparatively limited in human AS, the data also seem to support a similar bone marrow-derived pluripotent progenitor or early endothelial progenitor as a cell of origin in AS (69). A recent whole exome sequencing study of 20 HSA patients, and 30 more in a following study, identified multiple driver mutations shared with AS, including NRAS, PLCG1, PIK3CA and TP53 mutations with net activating effects on both the PI3K and MAPK signaling pathways (70, 71). Another recent study utilizing whole exome and RNA sequencing from a large cohort of Golden Retriever and mixed breed dog HSA showed additional shared molecular drivers including CDKN2A/B deletions and VEGFA, KDR, and KIT gain-of-function mutations (72). The driver mutations of KDR, TP53 and PIK3CA collectively identified across these three canine studies were also the primary recurrently mutated genes observed in an analysis of 47 human AS tumors by Painter et al. (73) Molecular data from both human AS and canine HSA increasingly support the presence of distinct molecular and functional tumor subtypes, which likely reflect differences in both cell-intrinsic mutations and influences from the tumor microenvironment and may have implications for prognosis and personalized treatments (68, 70, 74). Given the commonalities between HSA and AS within these proposed molecular subtypes, molecular subtyping may prove a promising means for overcoming disparate nomenclature schemes and enhancing translation of research between canine HSA and human AS.

### Stromal soft tissue sarcomas

In canine patients, multiple histologic subtypes of sarcomas arising from cutaneous and subcutaneous connective tissues are grouped under a shared grading system and are referred to collectively as soft tissue sarcomas (STS) (75, 76). Despite their distinct phenotypes and histogenesis, certain tumor subtypes within this grouping have very similar microscopic features, and immunohistochemical testing is typically required for their definitive diagnosis. Grade and mitotic index have been shown to be prognostic for these tumors, but the clinical significance of subtype in canine STS is less clear (75–78) and thus subtyping is frequently not pursued given the added time and client cost involved. In people, the term soft tissue sarcoma refers more broadly to all sarcomas arising from non-bony tissues and includes multiple sarcoma subtypes which are specifically excluded from the canine STS grouping due to their overall more aggressive biologic behavior in dogs (75, 77, 78). Furthermore, classification of human soft tissue sarcomas is increasingly defined by molecular and immunochemical testing not routinely available in veterinary medicine (18). Thus, facilitating more granular subtyping of canine STS, as well as clarification of the discordant veterinary and medical nomenclature schemes will be necessary prerequisites for fully leveraging this diverse collection of canine tumors in future pre-clinical and clinical research. Utilization of whole exome/genome and RNA sequencing technologies for comprehensive identification of cancer somatic variants, gene fusions, and transcriptomic signatures in canine STS should be a priority of the field to address this gap and better define histogenesis and molecular subcategories of these canine tumors which could inform human research. In this review, we will use the term stromal STS (sSTS) to collectively refer to the subset of human soft tissue sarcomas with histotypes corresponding to those in the canine STS grouping.

While the general histologic features are similar between canine STS and human sSTS the distribution of histologic subtypes varies between these species. In dogs, fibrosarcoma, peripheral nerve sheath tumors, and perivascular wall tumors are most common among STS for which subtype is reported. In contrast, liposarcoma and undifferentiated pleomorphic sarcoma are the most commonly reported sSTS in people (79, 80). As in canine STS, grade also appears to be strongly predictive of clinical outcome in human sSTS (80). Treatment principles for canine STS and human sSTS are similar, with wide surgical resection with complete histologic margins pursued as the primary treatment of choice in patients of both species (79–83). Adjuvant radiation and chemotherapy are also used with variable reported benefit (81–86).

Molecular characterization of canine STS represents an area where increased focus could overcome challenges posed by differences in current classification and nomenclature schemes to facilitate inclusion of these tumors in translational studies. For example, dermatofibrosarcoma protuberans in people is characterized by a pathognomonic COL1A1-PDGFB gene fusion (87), and though this fibrosarcoma subtype is not recognized in canine patients, RNA-seq analysis of a dermatofibrosarcoma protuberans-like tumor from a dog revealed an equivalent COL3A1-PDGFB fusion suggesting the presence of an analogous canine tumor (88).

The genetic landscape of human malignant peripheral nerve sheath tumors (MPNST) reveals recurrent themes of Ras/Raf pathway activation and impairment of DNA repair and P53 regulation resulting from mutations in NF1 (a hallmark of the inherited disorder neurofibromatosis type 1 which confers increased risk of MPNST), BRAF and CDKN2A/B (89–91). Limited genetic characterization of canine PNST indicate that a subset also have a correlate to the activating BRAFV600E mutation described in the human tumor (92), and alterations to the P53 regulatory system in the form of P35 mutations or MDM2 amplification have also been described in PNST in dogs (93). Interestingly, chromosomal rearrangements resulting in loss of heterozygosity in the CDKN2A/B cluster region similar to the alterations in this region in human MPNST have also been documented in two canine STS described as poorly differentiated fibrosarcomas (94). The authors in this study do not state what modalities beyond light microscopy were used in subtyping, and given significant microscopic overlap between canine PNST and fibrosarcoma, it is possible that these tumors may also represent PNST with a similar mutational profile to human MPNST.

Liposarcoma is an uncommonly reported STS subtype in dogs, but one for which histologic classification closely parallels human classification. Several immunohistochemical studies in canine liposarcoma demonstrate some similarities with the human disease, including overexpression of MDM2 in well-differentiated and de-differentiated variants. However these studies fail to provide evidence for other histotype-characteristic genetic alterations used in the diagnosis of myxoid and pleomorphic liposarcoma variants in people (95, 96). More complete genomic characterization of canine liposarcomas and other tumors within the canine STS categorization would be beneficial to better define their potential comparative applications.

### Other soft tissue sarcomas

Other rarer sarcoma subtypes occurring in dogs including gastrointestinal sarcomas (gastrointestinal stromal tumors (GIST) and leiomyosarcomas) and rhabdomyosarcoma, require further investigation but may also have potential applications as models for human disease. GIST and leiomyosarcoma are primary gastrointestinal sarcomas which account for only around 1% of GI malignancies in people (97, 98) but are reported to comprise up to 30% of all gastrointestinal neoplasms in dogs (99). These tumors are nearly indistinguishable microscopically, and prior to the routine use of immunohistochemistry for gastrointestinal biopsies, many GIST in both species were misdiagnosed as leiomyosarcomas (100–102). GIST and gastrointestinal leiomyosarcoma exhibit a range of biologic behavior, but distinction between these tumors is clinically important in people as activating mutations in exon 11 of the *c-kit* gene are implicated in the pathogenesis of many GIST and contribute to remarkable sensitivity of these tumors to tyrosine kinase inhibitors (103). As with many other sarcomas, cytogenetic data for canine gastrointestinal sarcomas are limited, but analogous activating *c-kit* mutations have been identified in canine GIST (104). Limited patient data has also demonstrated clinical benefit of the KIT-targeted tyrosine kinase inhibitor toceranib in canine GIST suggesting shared oncogenic mechanisms and therapeutic sensitivities that may make canine GIST a useful model for the human tumor.

Rhabdomyosarcoma refers to a group of malignant mesenchymal neoplasms which exhibit varying degrees of skeletal muscle differentiation. These neoplasms are rare in the veterinary literature, though this apparent low prevalence may be partially due to diagnostic challenges posed by the extreme variation in phenotype, age of presentation, and cellular morphology in these tumors (105). In people, rhabdomyosarcoma is the most common soft tissue sarcoma affecting children and teens, and over 50% of cases are diagnosed in children under 10 years of age (106). Similarly, and in contrast with the previously discussed sarcomas, canine rhabdomyosarcoma is also a disease primarily of young and adolescent animals (105). Canine rhabdomyosarcomas are classified using a scheme that is derived from human pathology, but which utilizes only histologic appearance and does not incorporate the immunohistochemical and molecular diagnostics that are now standard in subclassification of rhabdomyosarcomas in people. Based on this histologic classification scheme, the distribution of rhabdomyosarcoma subclasses in the dog appears similar to people (105, 106), suggesting that dogs with rhabdomyosarcomas may represent a yet largely untapped patient population which shares characteristics with the human rhabdomyosarcoma patient population. We recently published a small retrospective study on canine rhabdomyosarcoma which optimized immunohistochemical labeling for the myogenic transcription factors myogenin and MyoD1 in these tumors. This study provides a resource which may increase identification of these tumors in the canine population for future comparative biology investigations (107). To date only one cytogenetic examination has been performed on a single cell line derived from a canine pleomorphic rhabdomyosarcoma, which did not reveal any cytogenetic abnormalities (108). Further genomic characterization of canine rhabdomyosarcoma is needed to determine if the molecular pathogenesis of these tumors is similar to their human counterparts, with particular attention to the presence of PAX3/7-FHKR gene fusions, which are a hallmark of the more aggressive alveolar rhabdomyosarcoma subclass in people and have both diagnostic and prognostic utility (109–111).

## Considerations for co-clinical trial approaches in canine sarcoma patients

The conception and implementation of co-clinical trials approaches in translational oncology research has significantly expanded over the last decade. This expansion has been driven by technological advances which continue to provide an ever-increasing understanding of the mutational landscape and key genetic drivers of human cancer types. Our expanded understanding of this genetic complexity has led to an increasingly granular stratification of patients with a common cancer histology into diverse molecular subtypes. Personalized medicine through tailored therapeutic approaches targeting each individual patient’s actionable molecular drivers will no doubt led to additional clinical advances. However, the breadth and depth of these advances, and the time it takes to achieve them, continues to hinge entirely on effective pre-clinical modeling of the *in vivo* biological complexity of human tumors.

In part, this effective pre-clinical modeling has been achieved through significant advancements in 1) accessibility and feasibility of obtaining, expanding, and utilizing patient-derived xenograft (PDX) mouse models, and 2) insightful development and refinement of compelling genetically engineered mouse models (GEMMs) of human cancers. These pre-clinical *in vivo* models allow an almost exact recapitulation of treatment protocols for human clinical trial cancer patients in their GEMM or PDX counterpart. This type of mirrored approach can inform strategies and biomarkers for patient stratification, lead to the identification of mechanisms of *de novo* and acquired drug resistance, and rapidly evaluate drug combinations that overcome this resistance. For certain tumor types, the success of this approach has surmounted increasing cynicism of the basic and clinical oncology research communities around small animal pre-clinical cancer models. Still, these models are not without inherent challenges and disadvantages. PDX models require the use of immunodeficient mice, thus failing to recapitulate tumor-immune interactions and evolution and precluding investigation of anti-metastatic or immunomodulatory therapies. GEMMs alleviate some of these issues *via de novo* tumor development in the presence of an intact immune system, subsequent establishment of a microenvironment with tumor-host interactions, and in some models, spontaneous metastasis, but GEMMs can be limited in the number of concurrent mutations, limiting their ability to fully recapitulate the molecular heterogeneity of human tumors (112, 113).

**Figure 2:**
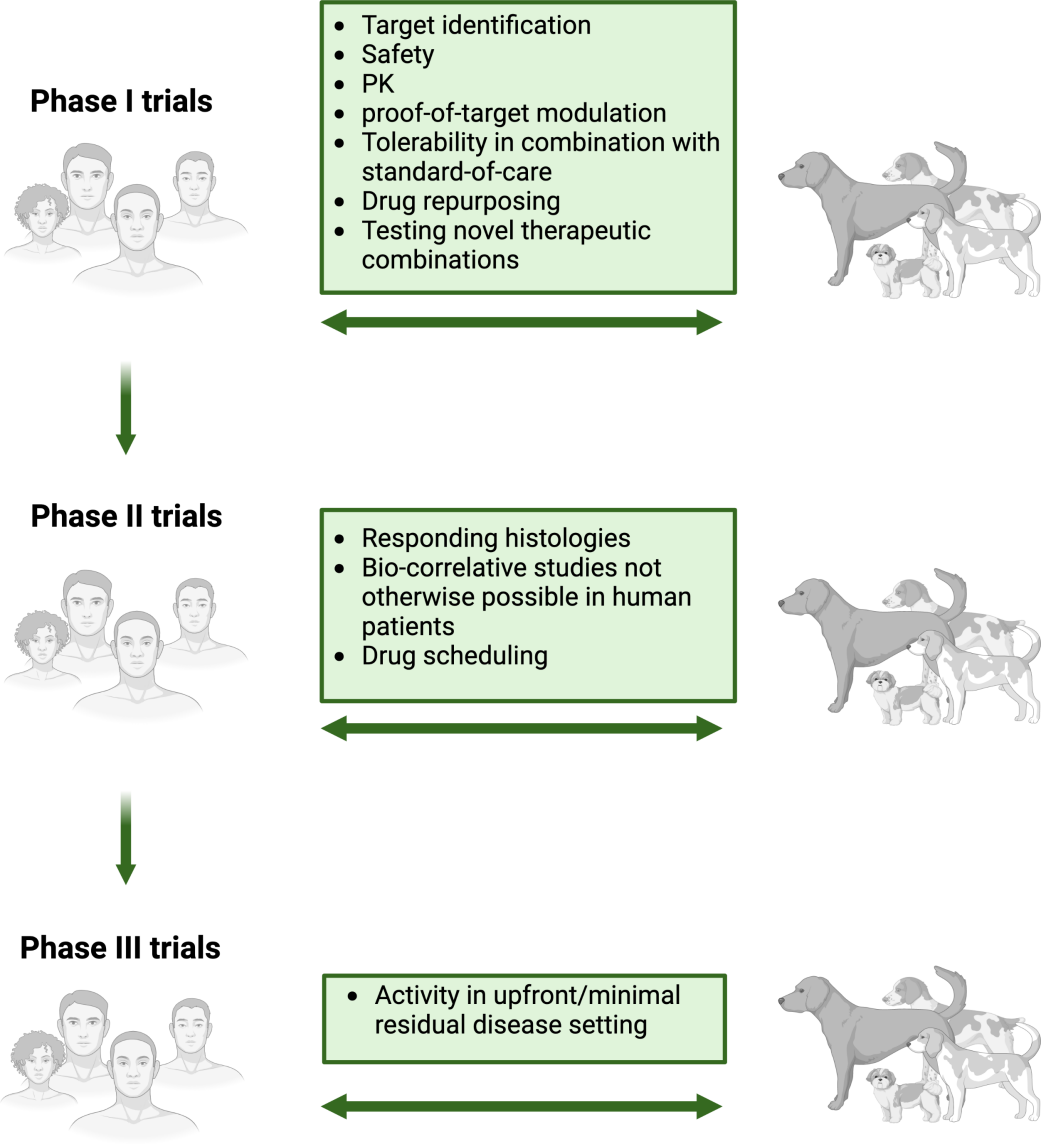
Integrating canine sarcoma trials in human oncology drug development. Considerations for the various types of clinical and biological data that can be generated through trials in dogs with spontaneous sarcoma and where in the human clinical trial pipeline integration of these data may be informative. Created with BioRender.com.

Spurred from these challenges is an increasingly renewed interest in employing a similar comparative dog-human co-clinical approach (**Figures 1**, **2** and **Table 1**). This approach has already proven to hold translational value and has the potential to fill some of the gaps associated with other pre-clinical models. Historically, the field of comparative oncology has provided the blueprint for the successful utilization of naturally occurring diseases in companion animals to conduct informative translational research. Clinical trials in dogs with spontaneous tumors were being conducted as early as the mid 1970s in pioneering bone marrow transplant studies by Storb and colleagues (114, 115). Other notable past advancements specific to sarcomas include development of human limb-sparing surgical techniques in dogs with osteosarcoma (116), and clinical evaluation of the biologic response modifier and macrophage immune stimulant liposomal muramyl tripeptide phosphatidyl ethanolamine (L-MTP-PE) in dogs with osteosarcoma by MacEwen et al., which provided key data supporting Phase II and Phase III trials of this compound in human patients (117). A more contemporary example of the dog-to-human pipeline includes drug repurposing studies for development of monocyte-targeted immunotherapy in dogs with metastatic osteosarcoma (34, 118), which has led to a Phase I trial in human patients (ClinicalTrials.gov Identifier: NCT03900793). **Table 1** provides a non-comprehensive list of active canine trials as a sampling of the intensive ongoing comparative oncology efforts to accelerate sarcoma therapeutic development. Nonetheless, leveraging dogs with naturally occurring tumors also presents its own unique set of barriers and considerations. These include competing trials for highly investigated tumor types like OS, which can result in bottlenecks in patient enrollment and subsequent failure to meet the desired timelines of funding sponsors, both industry and academic investigator alike. A lack of canine species-specific reagents is another gap which limits the ability of the comparative oncology field to provide more valuable bio-correlative data to pharmaceutical sponsors besides just clinical outcome (**Figure 1**). Moreover, the type of perfectly matched therapeutic mirroring that can be effectively accomplished between mouse and human patient is precluded by several hurdles in our canine patients. Many of the investigational drugs being screened in human patients have not undergone extensive toxicity and pharmacokinetic studies in dogs. If they have, these have often been completed by private pharmaceutical companies, and thus the necessary safety and dosing data required to design a canine-human co-clinical trial is often not publicly available or is entirely unattainable. While dose finding studies of investigational therapeutics are often conducted in dogs, this necessary prerequisite alone can undermine the time and cost efficiency gained from employing the drug in a co-clinical trial type approach. Furthermore, purely from a body size standpoint, obtaining these investigational therapeutics for co-clinical trial studies in dogs can be financially prohibitive when compared to their murine counterpart, if an industry collaboration is not established.

**Table 1:** Ongoing canine sarcoma trials that can inform human studies.

Canine Sarcoma Type	Therapeutic Agent/Molecular Target	Study Objective	Reference
Any	Ladarixin - oral, small molecule CXCR1/2 inhibitor	To determine whether co-administration of ladarixin can reduce chemotherapy-associated side effects.	https://vet.tufts.edu/clinical-trials
Primary appendicular osteosarcoma	Losartran/Toceranib/Ladarixin - Triple drug oral small molecule therapy targeting CCR2 monocytes (losartan), multiple kinases (toceranib), and CXCR1 and 2 (ladarixin)	To determine whether this triple drug oral immunotherapy combination given in the neo-adjuvant and adjuvant setting demonstrates equivalent clinical benefit (defined by progression-free and overall survival) to current adjuvant carboplatin chemotherapy.	https://www.csuanimalcancercenter.org/current-clinical-trials/ https://vet.tufts.edu/clinical-trials
Hemangiosarcoma	Not Applicable	Development and validation of the utility of plasma cell-free tumor DNA for early detection and genomic characterization of hemangiosarcoma.	https://vet.tufts.edu/clinical-trials
Primary appendicular osteosarcoma	Verdinexor - oral inhibitor of the nuclear export function of XPO1	To determine the safety and tolerability of verdinexor in combination with carboplatin chemotherapy.	https://www.vet.cornell.edu/hospitals/clinical-trials-0
Primary splenic hemangiosarcoma	Copanlisib - pan-class I phosphoinositide 3-kinase (PI3K) inhibitor	To determine the safety and efficacy of single agent PI3K inhibition for treating hemangiosarcoma.	https://www.vet.upenn.edu/research/clinical-trials-vcic/all-clinical-trials
Osteosarcoma and high grade soft tissue sarcoma	BG34-200 (200 kDa oat-derived β-(1-3)-(1-4)-glucan) - CD11b targeting myeloid cell immune stimulant	To determine the safety and efficacy of BG34-200.	https://ccr.cancer.gov/comparative-oncology-program/trials
Primary osteosarcoma	Novel near infrared imaging agent	Intraoperative detection of osteosarcoma surgical margins for improved success rate of limb-sparing surgery.	https://www.vet.upenn.edu/research/clinical-trials-vcic/all-clinical-trials
Splenic hemangiosarcoma	Propanolol - nonselective beta adrenergic antagonist and repurposed immune modulator via targeting of myeloid-derived suppressor cells	To determine whether propranolol in combination with standard of care doxorubicin chemotherapy can improve clinical outcome.	https://www.vet.upenn.edu/research/clinical-trials-vcic/all-clinical-trials
Soft tissue sarcoma	NF-κB essential modulator (NEMO)-binding domain (NBD) peptide inhibitor of NF-κB	To evaluate NF-κB target modulation and associated cytotoxicity.	https://www.vet.upenn.edu/research/clinical-trials-vcic/all-clinical-trials
Primary appendicular osteosarcoma	Vismodegib - hedgehog pathway inhibitor via competitive inhibition of Smoothened (SMO)	To evaluate safety and target modulation in combination with surgery and carboplatin adjuvant chemotherapy.	https://www.csuanimalcancercenter.org/current-clinical-trials/
Lung metastatic osteosarcoma	Defactinib - focal adhesion kinase (FAK) inhibitor	To determine the safety, efficacy, and proof-of-target modulation of defactinib in combination with losartan and toceranib in dogs with metastatic osteosarcoma.	https://www.csuanimalcancercenter.org/current-clinical-trials/
Lung metastatic osteosarcoma	Inhaled recombinant human IL-15	To determine the safety and efficacy of inhaled IL-15 in combination with doxorubicin standard of care.	https://ccr.cancer.gov/comparative-oncology-program/trials

Thus, enhancing engagement and collaboration between academic physician-veterinarian research teams and the pharmaceutical industry is something that must be increasingly addressed in the comparative sarcoma research community. One potential solution to this pressure point is the National Cancer Institute’s (NCI) Cancer Therapy Evaluation Program (CTEP). The long-established NCI Comparative Oncology Trials Consortium (COTC) could work internally with the NCI Division of Cancer Treatment and Diagnosis to develop a secondary function wherein COTC could enter into collaborative agreements with pharmaceutical companies similar to those of CTEP. This could allow COTC, perhaps through CTEP's already established “Non-clinical Use Request” avenue, to supply academic investigators of human-canine veterinary cancer center consortium institutions (e.g. The V Foundation Canine Oncology Research Consortium – CORC) with investigator brochure canine pharmacokinetic and toxicity data and the desired investigational agent or active pharmaceutical ingredient for compounding.

Despite these challenges, co-clinical trial designs in dogs with spontaneous sarcomas can still significantly aid in pharmacokinetic and pharmacodynamic based vetting of pre-clinical therapies (**Figure 2**). Co-clinical trials may also provide equal or greater efficiency and utility in 1) addressing the biology underlying mechanisms of therapy response and sarcoma progression and metastasis, and 2) in “high-risk high-reward” biomarker evaluation or combination therapy clinical approaches that would otherwise not be attempted in human patients, no matter how positive the pre-clinical supportive data is. These suggestions are not to say that investigations leveraging dogs as a surrogate for human sarcomas should not be supported by strong pre-clinical data from cellular and small animal models. Nor are they meant to undermine the undeniable value of the canine patient for preclinical assessment of safety, efficacy, pharmacokinetics, and pharmacodynamics of novel therapeutic candidates, or in correlative studies on parallel evaluation of biologic responses and predictors to the therapeutic actions of these candidates.

A prime example of the type of “high-risk high-reward” investigations that may best leverage dogs with spontaneous sarcoma is metastasis biology research in osteosarcoma. Metastasis accounts for greater than 90% of all cancer-related mortality (119), and in OS, roughly 30% of patients with localized disease at diagnosis die within 5 years due to metastasis (120). Lung metastasis accounts for up to 90% of OS recurrence, occurring on average 1.6 years after diagnosis and portending an unacceptable 20% 5-year survival (21, 121, 122). Like their human counterparts, dogs with naturally occurring sarcomas develop metastasis spontaneously over a still protracted but more chronologically relevant scale and after successful first-line therapy for their primary tumor. Thus, canine OS patients provide an incredible window of opportunity to conduct high risk clinical studies in the setting of pre-/micro-metastatic disease (**Figure 2**), an otherwise ethically and technically prohibitive endeavor in their human counterpart (8, 16, 123–126). These studies can inform new biomolecular imaging techniques, screen for early and more robust biomarkers of metastasis development, and test anti-metastatic drug strategies in the adjuvant/minimal residual disease setting. The latter is increasingly important as many anti-cancer drugs screened in early phase clinical trials are often evaluated in the setting of gross metastatic disease, despite their initial pre-clinical efficacy signal first being validated in mouse models of microscopic/minimal residual.

Stephen Paget’s 1889 “seed and soil” hypothesis on metastatic organotropism continues to foresee one of the most intensive areas of cancer research over the last two decades (127–130). Despite this intensive focus on the mechanisms of “pre-metastatic niche” formation in distant organs, mice are essentially the singular model for this area of investigation, and there remain significant obstacles to evaluating the clinical importance of pre-metastatic niche formation in human cancer patients. Nonetheless, experimental data from mouse models has demonstrated the lung’s unique permissiveness to pre-metastatic priming, highlighting it as an archetype of metastatic organotropism for many solid cancers. Retrospective studies estimate pulmonary metastases occur in 20-54% of patients who die from extra-thoracic malignancies, and the lung is a top 3 metastatic site for 9 of the 18 most lethal cancers in the U.S (131). As summarized above, canine OS is an established, well-recognized, and translationally relevant animal model with the potential to rapidly accelerate discovery in human OS (8, 16, 123, 124). Additionally, the significantly high and early lung metastatic tropism of canine OS represents a spontaneous large animal model with significant potential to inform discoveries on basic mechanisms of lung metastasis which are fundamentally applicable across other tumor types. Given the uniquely strong tropism of OS cell dissemination to the lung in both humans and dogs, insights gained into the fundamental biology underlying lung metastasis development in OS from investigations in dogs are likely to provide a translational value equal to those comparative oncology trials solely focused on pre-clinical vetting of novel therapeutic candidates. Moreover, comparative studies addressing these more basic research questions could help to better address potential concerns regarding the predictability of the canine model for human (osteo)sarcoma.

While remarkable clinical and biological similarities of human and canine OS have been previously demonstrated, increasing knowledge of the genomic complexity in human OS obtained through investigations leveraging technological advances is beginning to widen the gap between these parallel patient populations. If not addressed, this widening gap in the molecular understanding of human vs. canine OS could undermine the predictability of the canine model for testing novel preclinical candidates more so than in studies focused on the fundamental biology of sarcoma progression and metastasis. However, work on addressing this knowledge gap has already begun through establishment of the NCI’s Integrated Canine Data Commons (https://caninecommons.cancer.gov/#/home), a centralized, open access repository of clinical and genomic data from spontaneous canine cancers, and by private biotechnology companies such as nanoString® who have made significant investments in canine comparative oncology research (**Figure 1**), such as development of the nCounter canine IO® panel, and canine cancer atlas GeoMX® spatial transcriptomics. Continued funding and expansion of the comparative oncology molecular toolbox over the next decade will surely close this gap and continue to illuminate key species similarities and differences in sarcoma biology. To this end, another mechanism to incentivize canine species-specific reagent development such as therapeutic and diagnostic antibodies could be continued engagement between private biotechnology companies and the academic investigators and core facilities which are members or supporting laboratories of the NCI Comparative Oncology Trials Consortium or PRECINCT (PRE-medical Cancer Immunotherapy Network Canine Trials; https://www.precinctnetwork.org/). Through an NIH-hosted workshop, these groups of comparative oncology investigators could provide a list of prioritized canine targets for which antibody reagents are needed. Subsequently, working through the channels of the Comparative Oncology Program or Canine Cancer Immunotherapy Network, NCI could produce a Small Business Innovation Research (SBIR) request for proposals for companies to produce these antibody reagents. Once produced, competitive supplemental funding could then be provided to NCI PRECINCT members who submit detailed proposals for validating the specificity and functionality of these antibodies in a variety of immune assays.

## The veterinary clinician-scientist pipeline

Continued efforts in diversification of our cancer research workforce training infrastructure is an indispensable prerequisite for enhancing the quantity and quality of comparative sarcoma research. To be successful in this approach, the next generation of cancer researchers must include veterinary biomedical scientists with training backgrounds in medical, surgical, and radiation oncology, pharmacology, pathology, and immunology (**Figure 1**). In addition to their veterinary training, these individuals need an outlet, through partnerships with medical schools, to participate in comparative training opportunities. In some cases, these opportunities exist in the form of clinical trials design training and MS certification programs, non-MD-restricted molecular genetics fellowships, post DVM residency training with a specific focus on lab animal medicine or pathology, and NIH Clinical and Translational Science Awards (CTSA) sponsored training and partnerships such as TL1 training grants and CTSA One Health Alliance (COHA) training fellowships (https://www.ctsaonehealthalliance.org/). Additional opportunities in areas such as advanced molecular imaging, cross disciplinary training in human molecular pathology and human investigative new drug trial design would allow veterinary clinician scientists to bring these skills back to our patients and better inform comparative oncology trial design and laboratory correlative studies to maximize extraction of data from these trials and increase their impact on clinical sarcoma patient management.

Sustaining and bolstering cross-veterinary and -medical institutional support in the form of expanded financial and faculty training investment into specialized and clinically relevant comparative and translational biomedical research fellowship training programs for veterinary scientists would provide more than just a pipeline of well-trained, diverse clinician scientists to foster co-clinical trial approaches in large animal models. Specifically, these fellowship programs would be separate from the already successfully established, and in some cases National Institutes of Health training grant funded, DVM-PhD and post-DVM residency/PhD programs established at veterinary schools across the USA. Instead, these fellowships could more closely mirror MD post-residency clinical fellowships programs, which often contain a multi-year research intensive component and in many cases lead the trainee to pursue further post-doctoral research following fellowship completion. In veterinary medicine, establishment of these fellowship programs would serve to further develop and fortify a more integrated veterinary and medical oncology research landscape from its current partially siloed state, into one that effectively blends and aligns the comparative oncology trials work of veterinary clinicians and scientists with their human colleague counterparts. Veterinary clinicians and scientists completing these fellowships would likely emerge with strong established collaborations with their human oncology research counterparts, a key benefit that circumvents an otherwise not infrequent barrier for junior veterinary clinician scientist faculty looking to establish independent comparative oncology research programs.

Similar to their medical counterparts, veterinary clinician-scientists are invaluable members of increasingly necessary multi-disciplinary cancer research teams. If and when these types of translational research fellowships are established, recruiting, retaining, and growing the number of clinically trained veterinarians entering into these programs would in part require a paradigm shift in how veterinary medical institutions themselves value these post-DVM trainees. Based on the latest American Veterinary Medical Association (AVMA) compensation report, veterinarians entering academic residency or PhD programs immediately after graduation would likely receive 40-50% of the starting salary of their fellow classmates entering into private clinical practice despite comparably high educational loan burdens. When considering post-residency DVMs entering PhD or the fellowship programs proposed here, this compensation gap exponentially widens. Post-residency academic fellowship programs that provide compensation at the NIH-specified post-doctoral stipend levels, regardless of whether or not the program holds, or the individual successfully competes for, an NIH institutional training grant, would provide the necessary institutional commitment needed to broaden the veterinary clinician-scientist pipeline.

There exists already a strengthening network of key academic, government, and non-profit stakeholders with an expanding intellectual and financial investment in comparative sarcoma research. For example, opportunities are already arising to share ideas and data from members of canine clinical trials and comparative oncology research consortia such as the Comparative Oncology Trials Consortium, the Consortium for Canine Comparative Oncology (c3o) between Duke Cancer Institute and North Carolina State University College of Veterinary Medicine, and NCI PRECINCT (which was created through the aforementioned NCI Cancer Moonshot funding opportunities), with that of their human counterparts such as the Children’s Oncology Group Osteosarcoma Biology and Bone Tumor Steering Committees. These collaborative opportunities occur through 1) focused comparative oncology scientific sessions at national meetings, 2) joint national meetings between canine and human cancer moonshot grant awardees, 3) funding opportunities requiring veterinary and medical scientist co-PI submissions (*V* foundation for Cancer Research), and 4) inclusion of members of both groups in monthly grant progress and update meetings of these consortia. Additionally, non-profit groups such as Ethos Discovery, the Osteosarcoma Institute, and Make It Better (MIB) Agents are fully invested in engaging academic investigators, leveraging their collaborative networks, and raising funds to support both human and canine sarcoma research.

## Conclusion

Although clear gaps in the comparative molecular characterization of the canine sarcoma types reviewed herein remain, these temporary limitations should not preclude the cancer research community’s embracement of these valuable translational models. Leveraging dogs with spontaneous sarcomas in co-clinical trial approaches will undoubtedly provide a valuable and complementary source of high predictive value data to inform clinical practice, and ultimately, improve both human and canine sarcoma patient outcomes. Fully realizing the potential of this comparative approach requires fortifying veterinary and human medical oncology research partnerships alongside continued and enhanced investment in training the next generation of diverse, cross-disciplinary cancer researchers.

## Author contributions

MK, LH, LA, AM, ML and DR all drafted and edited the manuscript. MK and DR generated the figures and table. All authors contributed to the article and approved the submitted version.
